# Continuous Blood purification on Influenza-Associated Neurological Disease in children: a retrospective cohort study

**DOI:** 10.1186/s12879-021-06265-7

**Published:** 2021-07-10

**Authors:** Jingwen Ni, Kenan Fang, Zhe Zhao, Zhiyuan Wang, Qian Huang, Lele Li, Guiying Yang, Huizi Guo, Xiaoyang Hong, Shujun Li

**Affiliations:** 1 Pediatric intensive care unit, Luoyang Maternal and Child Health Hospital, Luoyang, China; 2 Pediatric intensive care unit, Department of Pediatric, PLA General Hospital, Beijing, China; 3 Pediatric intensive care unit, The second school of Clinical Medicine, Southern Medical University, Guangdong, China; 4 Pediatric intensive care unit, The First Affiliated Hospital of Xinxiang Medical University, Xinxiang, China

**Keywords:** Continuous blood purification, Influenza, Children, Neurological complications, Retrospective cohort study

## Abstract

**Background:**

Due to lack of proven therapies, we evaluated the effect of CBP on Influenza-Associated Neurological Disease in children.

**Methods:**

A single-center, retrospective, cohort study was conducted in Luoyang, Henan province, China from January 2018 to January 2020. Children (<18 years) with influenza-associated neurological disease were enrolled in the study. Children with indications for CBP and parental consent received CBP (Continuous Blood purification), while others received maximal intensive care treatment because of the absence of parental consent. The outcomes of the CBP and non-CBP groups were compared. Categorical variables were presented as percentage and compared by Chi-square test. Continuous variables were expressed as median (interquartile ranges) and compared with non-parametric independent sample test. Statistical analyses were carried out by SPSS (version 26.0) and p < 0.05 (2 tailed) was considered to be statistically significant.

**Results:**

30 children with influenza-associated neurological disease were recruited to the study. 18 received CBP and the other 12 received maximal intensive care. There were no differences between CBP and non-CBP children in age, sex, body weight, type of influenza virus, neurological complications, Glasgow score, PIM-2 score and PCIS at admission (p > 0.05). The inflammatory factors (CRP, PCT and IL-6) of 30 cases were tested at admission and after 3 days of admission. In the CBP group, there was a significant decrease in IL-6 levels at 3 days of admission (p = 0.003) and a decrease in CRP and PCT levels, but no significant difference (p > 0.05). In the non-CBP group, there were no significant difference on levels of CRP, PCT and IL-6 at admission and 3-day of admission (p > 0.05). The 28-day mortality was significantly lower in the CBP group compared with the non-CBP group (11.11% vs. 50%, p = 0.034).

**Conclusions:**

CBP definitely reduces IL-6 levels significantly. We did find that the survival rate of patients in the CBP group was improved. But we don’t know if there is a relationship between the reduction of IL-6 levels and the survival rate. Trial registration: http://www.chictr.org.cn/index.aspx(ChiCTR2000031754).

## Background

Seasonal influenza epidemics cause between 3–5 million cases of severe illness and approximately 290,000 to 600,000 deaths every year in the world [1]. Most people are susceptible to influenza, which is generally a self-limiting disease. However, individuals in high risk groups, such as infants, can develop severe complications. These complications include pneumonia, neurological complications and even multi-organ dysfunction. Although pneumonia is the most common complication in children, neurological disease is one of the most severe complications [2]. The actual incidence of neurological complications in children with influenza infection is unclear. However, it is more common compared to adults, accounting for 1.7–15% of children hospitalized with influenza infection [3], and the burden of influenza-associated neurological disease (IAND) is higher in the pediatric population. In the United States and the UK series, children account for 73% and 84% of IAND cases, respectively [4]. The most frequently reported complications are as follows: seizures, encephalopathy, Reye syndrome, acute disseminated encephalomyelitis, myelitis, Guillain-Barre syndrome, acute necrotizing encephalopathy (ANE) and speech or movement disorders [3]. The prognosis of influenza-associated neurological diseases has been reported to vary [5, 6], with encephalopathy and acute necrotizing encephalopathy having the poorest prognosis. among which encephalopathy and acute necrotizing encephalopathy have the worst prognosis. The main cause of death is multiple organ failure, which usually occurs within 2 days of the occurrence of neurological symptoms, with a mortality rate of 31.8% [3]. In addition to supportive treatment, there is no effective treatment for the Influenza-associated neurological disease until now. The systemic inflammatory response syndrome after influenza virus infection has been shown to play an important role for neurological complications [7]. However, CBP can remove inflammatory factors and reduce the level of inflammatory factors in the serum of sepsis patients [8].

But, It is unknown whether the outcome and prognosis would be improved, if CBP is applied for removing inflammatory factors in influenza children with neurological complications. The aim of our study was to determine whether CBP has superior outcomes and prognosis in neurological complications in children with influenza compared to non-CBP.

## Methods

### Study design

A single-center, retrospective cohort study was conducted in Henan, China, from January 2018 to January 2020. A total of 545 pediatric patients (<18 years old) were admitted to PICU of Luoyang Maternal and Child Health Hospital for the diagnosis of influenza. Among these children, the cases with neurological complications were enrolled into this study. The inclusion criteria include:129d-18y; 2 The samples were collected from nasopharynx swabs, and the antigen was detected by colloidal gold method. Among the 30 children, 26 children were positive for influenza antigen, while the other 4 children were negative. These 4 children are in the high epidemic season, and have close contact with influenza-like patients; in addition their clinical symptoms are in line with the performance of influenza. Therefore, 30 children were clearly diagnosed with influenza finally [9]. 3 The emergence or worsening of a neurological symptom, which could not be explained otherwise, in a patient with laboratory proven influenza [3]. 4 Respiratory support by ventilator. Exclusion standard: 1 The children who had received cardiopulmonary resuscitation before admission and had fixed dilated pupils, or unequal pupil size, or low EEG voltage (<5Hz); 2 The children who abandoned treatment within 72 hours of admission for family reasons; 3 There are only frequent convulsions, but no progressive aggravation of consciousness disorder during the course of the disease; 4 There are high risk factors for bacterial purulent meningitis such as rhinorrhea, middle ear deformity or skull base fracture; 5 The children who has basic brain disorders such as metabolic encephalopathy or epileptic encephalopathy.

### Clinical management

The routine treatment for all the children included: neuraminidase inhibitors such as Peramivir [9] against influenza virus, ventilator-assisted ventilation, mannitol dehydration to lower intracranial pressure, midazolam and sufentanil for sedation and analgesia to reduce oxygen consumption, nutritional support etc. We treated with gamma globulin, methyprednisolone and plasma exchange for acute necrotizing encephalopathy of children. Elevation of the head of the bed, aspiration of subglottic secretions, control of the colonization of the oropharynx and the digestive tract to avoid ventilator-associated pneumonia. The vital signs, including heart rate, respiratory rate, transcutaneous oxygen saturation was monitored, as well as the 24 hours intake and output per day. The children with CBP indications and the parents’ consent received CBP, and the others were treated with routine intensive care due to failure of parents’ consent.

The internal jugular vein or femoral vein were selected for catheterization in all children. Blood filters were used according to the children’s weight. The CBP treatment included PE and CVVHDF. On the first day and the second day, the plasma exchange was conducted firstly, with the plasma exchange of 60ml/kg/d. Standard volume CBP (25ml/kg/h) was performed after plasma exchange. The duration of treatment was $\geqslant $16 hours per day. Albumin, plasma or saline were selected as priming fluid before treatment according to body weight. For children less than 3kg, suspended red blood cells were selected as the priming fluid. For children with 3–15kg body weight, we selected albumin or plasma as priming fluid and saline for the children greater than 15kg. The right internal jugular vein was implanted with a double-lumen catheter for drainage and the left or right axillary vein with a 22G indwelling needle for blood return due to the limitation of vascular conditions in children under 2 months old. Double lumen catheters were implanted into internal jugular vein and / or femoral vein in children over 2 months old. The type of double lumen catheter was selected according to the diameter of vascular minor axis measured by superficial ultrasound probe. A 5F double lumen catheter was used for vessels with diameter ≤ 0.6cm, 7F double lumen for vessel between 0.6cm and 1.0cm and 11.5F for vessels with diameter over 1cm.

### Outcomes

The primary outcome was 28-day mortality at admission. Secondary outcomes included ICU stay, Glasgow score, PIM-2, PCIS, ventilator-time, hospitalization costs.

### Statistical methods

The outcomes were compared between CBP group and non-CBP group. Categorical variables were presented as percentage and compared by Chi-square test. Continuous variables were expressed as median (interquartile ranges) and compared with non-parametric independent sample test. Statistical analyses were finished by SPSS (version 26.0) and p < 0.05 (2 tailed) was considered to be statistically significant.

## Results

**Fig. 1:**
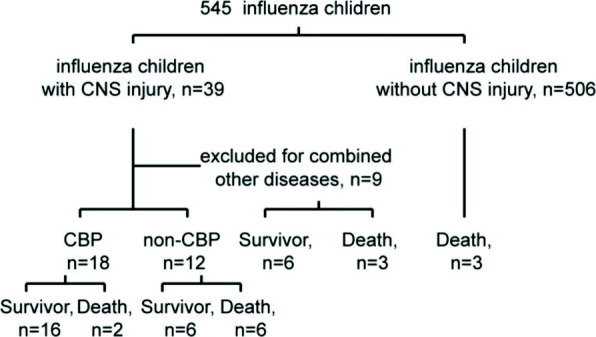
Workflow process to obtain influenza children with neurological complications

Working flow and basic information of influenza children in PICU. The study collected 545 children with influenza admitted to PICU from January 2018 to December 2019 for retrospective analysis. 39 of them had Influenza-associated neurological disease. 9 cases did not meet the inclusion criteria and were excluded. Among these 9 cases, 2 children received cardiopulmonary resuscitation before admission, after admission, the pupils were dilated and fixed or pupils were not large, and the brain voltage was lower than 5 Hz. One child was given up within 72 hours of treatment; 6 children gradually recovered their consciousness after admission and gradually recovered without receiving advanced life support such as ventilator-assisted ventilation.30 children were enrolled in this study. 18 received in CBP group with parents’ consent, and the other 12 were in non-CBP group due to the failure of parents’ consent (see Fig. 1).

The demographic characters and clinical information at admission were showed below. There were no differences between CBP and non-CBP children in age, sex, body weight, type of influenza virus, neurological complications, Glasgow score, PIM-2 score and critical ill score and the level of Inflammatory factors at admission (p > 0.05) (see Table 1).

**Table 1:** Demographic characteristics and clinical information at admission

Characteristics		CBP group (n=18)	Non-CBP group (n=12)	*p*
Age (median [IQR]) (m)^b^		19 (8.50-34.50)	20.5 (9.13-47.75)	0.932
Sex	Male^a^	9	5	0.722
	Female^a^	9	7	
Body weight (median [IQR])(kg)^b^		10 (7.65-14.13)	11 (8.13-14.25)	0.734
Type of influenza	A^a^	12	8	1.0
	B^a^	6	4	
Neurological complications	Encephalopathy^a^	10	7	1.0
	Encephalitis^a^	8	5	
Glasgow score (median [IQR])^b^		4.00 (4.00-5.25)	4.00 (3.00-5.00)	0.479
PIM-2 score (median [IQR])^b^		25.05 (18.40-78.63)	20.25 (17.25-70.05)	0.498
Critical ill score (median [IQR])^b^		74 (71.0-76.5)	81 (68.50-87.00)	0.349
Inflammatory factors	CRP(median [IQR]) (mg/L)^b^	14.35 (1.85-23.63)	7.95 (3.71-21.48)	0.983
	PCT(median [IQR]) (ng/ml)^b^	3.4 (0.28-20.50)	22.5 (5.13-38.75)	0.099
	IL-6(median [IQR]) (ng/ml)^b^	0.96 (0.42-2.40)	0.69 (0.31-1.335)	0.611

*CBP* Continuous blood purification, *IQR* Interquartile range, *PIM-2* Pediatric index of mortality 2, *CRP* C-reactive protein, *PCT* Procalcitonin, *IL-6* interleukin-6

^a^ *p*<0.05 The sex, type of influenza and neurological complications using the Chi-squared test

^b^ *p*<0.05 The age, Body weight, Glasgow score, PIM-2 score, Critical ill score, CRP, PCT, IL-6 using nonparametric tests/2 independent samples/Mann-Whitney *U* test

### Inflammatory factors after CBP

**Fig. 2:**
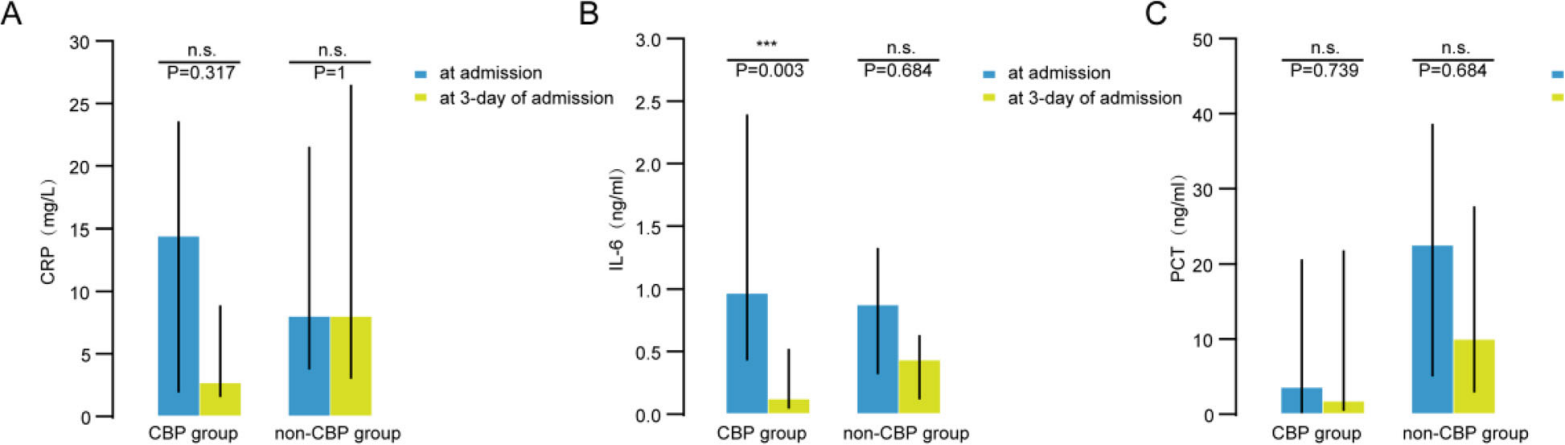
Statistical charts of the inflammatory mediators of CBP group and non-CBP group at admission and 3-day of admission. **A** Compared to 3-day of admission, statistics charts show decreasing of CRP in CBP group, but there was no significant difference (14.35[1.85,23.625], 2.700[1.500,8.968], p = 0.317). There was no difference of CRP in non-CBP group (7.950[3.705,21.475], 7.900[3.050,26.475], p = 1.0). **B** Compared to 3-day of admission, statistics charts show significantly decreasing of IL-6 in CBP group (0.955[0.423,2.400], 0.120[0.040,0.525], ***p = 0.003). There was no difference of IL-6 in non-CBP group (0.690[0.313,1.325], 0.435[0.123,0.628], p = 0.684). **C** Compared to 3-day of admission, statistics charts show decreasing of PCT in both in CBP group and non-CBP group, but there was no significant difference (CBP group: 3.400[0.278,20.500], 1.800[0.290,21.775], p = 0.739; non-CBP group: 22.500[5.125,38.750], 9.900[2.825,27.500], p = 0.684).

The level of CRP, PCT and IL-6 of serum in CPB group were lower than in non-CBP group at 3-day of admission, with no significant difference (p > 0.05). In CBP group, the level of IL-6 was significantly lower at 3-day of admission than at admission (p = 0.003), the level of CRP and PCT also decreased at 3-day of admission, but with no significant difference (p > 0.05). In the non-CBP group, there were no significant difference on levels of CRP, PCT and IL-6 between at admission and 3-day of admission (p > 0.05) (see Fig. 2).

### Outcomes

The 28-day mortality rate was significantly lower in the CBP group than in the non-CBP group (11.11% vs. 50.00%, p = 0.034). Glasgow score, PIM-2 score and PCIS also significantly improved in the CBP group. At the same time, the length of PICU stay was significantly prolonged, and the hospitalization costs were significantly increased (see Table 2). Two of the CBP-treated children were found to have hypothermia and severe hypophosphatemia, and two children had catheter puncture site bleeding as a result of early DIC during the course of the disease.

**Table 2:** Outcomes of CBP and non-CBP groups

Outcomes	CBP group (n=18)	Non-CBP group (n=12)	*p*
28-day mortality, n (%)^a^	2 (11.11)	6 (50)	0.034*
Glasgow score (median [IQR])^b^	7.00 (4.00-7.25)	3.50 (3.00-5.75)	0.021*
PIM-2 score (median [IQR])^b^	11.00 (7.78-22.55)	43.80 (9.28-85.03)	0.040*
Critical illness score (median [IQR])^b^	93 (88.00-94.00)	81 (77.00-93.50)	0.038*
PICU stay (median [IQR]) (d)^c^	19.00 (15.00-22.50)	10.00 (4.00-12.00)	0.008*
Ventilation time (median [IQR]) (d)^c^	7.50 (5.75-15.25)	5.50 (4.00-6.75)	0.060
hospitalization costs(median [IQR]) (US dollars)^c^	11350.67 (8670.36-16802.95)	6195.63 (3246.95-8000.03)	0.008*

*CBP* Continuous blood purification, *IQR* Interquartile range, *PIM-2* Pediatric index of mortality 2, *PICU* Pediatric intensive care unit

^*^ *p*<0.05

^a^ *p*<0.05 The 28-day mortality using the Chi-squared test

^b^ *p*<0.05 The Glasgow score, PIM-2 score, Critical ill score using Mann-Whitney U test

^c^ *p*<0.05 The PICU stay, Ventilation time, Hospitalization costs using nonparametric tests/compare medians across groups

## Discussion

We retrospectively analyzed 545 children with influenza in the PICU, 30 of whom developed neurological complications and were included in the study. Influenza infection is a serious threat to the health of children. In 2003–2004 influenza season, the average hospitalization rate was 36/100000 of American children, but up to 80% for children under 5 years of age [10]. A study on influenza-associated neurological diseases from Australia showed that seasonal influenza is an important cause of children’s neurological diseases in Australia. After extensive influenza vaccination, the mortality of children with influenza-associated encephalopathy was significantly reduced [11]. Data of influenza-associated encephalopathy reported through Japan’s National Infectious Disease Epidemiological Surveillance Database from 2010 to 2015 showed a total of 385 patients got with influenza-associated encephalopathy. The average incidence of influenza-associated encephalopathy in children and adults (age $\geqslant $18 years) was 2.83 and 0.19 per million, respectively. The median duration of fatal cases from the onset of influenza-associated encephalopathy to death was 1 day [12]. In China, the Beijing Children’s Hospital conducted an analysis of the cause of death of 19 children with influenza virus infection from November 2017 to April 2018, 8 children had flu-associated encephalopathy, and 7 of them died of flu-associated encephalopathy [13].

In addition to severe respiratory complications, which can rapidly progress to coma in some children, influenza-associated neurological complications are more severe in children and are the leading cause of influenza death [12, 14, 15]. Until now, the pathogenesis of influenza-associated neurological complications is still unclear. Autopsy of these patients showed necrosis and epithelial hemorrhage of thalamus and posterior cerebral cap of pons, pale myelin sheath in white matter of brain and cerebellum, and no clear obstruction of vascular endothelium and peripheral vascular edema [16]. Due to the acute onset of influenza-associated neurological diseases, severe brain dysfunction can occur quickly. The treatment time window is tight. Before the symptoms of nervous system appear, the children’s respiratory system disorder is not prominent or even no respiratory system performance, and lack of early warning indicators. Although immunization can reduce its incidence to a certain extent [11], most children are not actively vaccinated. Once the patient developed into an Influenza-associated neurological disease, treatment is extremely difficult, and some children develop cardiopulmonary failure within a short period of time. Many children have undergone cardiopulmonary resuscitation before admission, and showed pupil light loss or even brain herniation after admission, the brain failure will soon appear even if with active treatment. Even if they survived, some children will still have serious neurological complications. The mortality rate of acute necrotizing encephalopathy is 31.8%, and only 10% of patients make a full recovery, with many more surviving as neurological sequelae. The neurological sequelae recover very slowly and some patients received unacceptable long-term rehabilitation [17]. Therefore, effective treatment of influenza-associated neurological disease, especially acute necrotizing encephalopathy is a problem that we urgently need to solve. In this study, we found that the mortality of children in the CBP group was lower than that of the non-CBP group, and the neurological coma score (Glasgow score), PCIS score and PIM-2 score were improved compared with the non-CBP group. This suggests that CBP is associated with a reduction in mortality.

The pathogenesis of influenza-associated neurological disease is unclear. The current view is that influenza virus infection triggers a systemic inflammatory response syndrome, in particular, elevated cytokines (interleukin-6, -8, -10) are involved. The physiological states of IL-6 is knows to have a neuroprotective effect, but abnormal increase of IL-6 is found in serum of IAE patients with severe disability or death with serious adverse prognosis [17, 18]. This is consistent with the fact that IL-6 plays a more obvious role in nervous system diseases related to influenza reported in the literature [4]. Some people that the IL-6 overexpression subsequently induces or contributes to NMDA-mediated neurotoxicity, in pathological states [19]. Therefore, the elevated cytokines (interleukin-6, -8, -10), caused by systemic inflammatory response syndrome triggered by influenza virus infection, have been hypothesized to play an important role in the pathogenesis of neurological complications [14, 20]. However, there is no effective therapy for the “system cytokine storm” caused by influenza virus. Corticosteroid is the most commonly used and the effect is still controversial. The current treatment for influenza-associated neurological disease is symptomatic treatment and immune regulation. Kawashima [21] reported three children with influenza encephalopathy recovered from influenza after hormone and plasma exchange. The CBP model in this subject includes PE and CVVHDF, which can remove inflammatory factors between 0.5k-60 kDa from serum. IL-6 molecular weight is 24.385kD. In this study, the level of inflammatory factors,including CRP, PCT and IL-6, reduced in the CBP group after blood purification. Among them, the level of IL-6 decreased more significantly.

CBP can definitely reduce IL-6 levels significantly, and the survival rate of patients in the CBP group was improved. However, since this study is a retrospective study, all data are dependent on the records in the previous database. What’s more, due to the effective number of samples in this study, we don’t know if there is a relationship between the reduction of IL-6 levels and the survival rate. So, the mechanism and efficacy of CBP therapy in the treatment of Influenza-associated neurological disease require further research and discussion.

## Conclusions

CBP can definitely reduce IL-6 levels significantly. We did find that the survival rate of patients in the CBP group was improved. But we don’t know if there is a relationship between the reduction of IL-6 levels and the survival rate.

## Data Availability

The datasets used and/or analyzed during the current study are available from the corresponding author on reasonable request.

## Abbreviations

CBP: Continuous Blood purification
CRP: C-reactive protein
PCT: Procalcitonin
IL-6: Intereukin-6
PIM-2: pediatric index of mortality-2
PCIS: Pediatric critical illness score
CVVHDF: Continuous venovenous hemodiafiltration
PE: Plasma exchange
DIC: Disseminated intravascular coagulation
TNF- *α*: Tumor necrosis factor *α*
